# Deep learning analysis of MRI to assess rectal cancer treatment

**DOI:** 10.3389/fonc.2025.1643852

**Published:** 2026-02-09

**Authors:** Heather M. Selby, Ashley Y. Son, Vipul R. Sheth, Todd H. Wagner, Erqi L. Pollom, Arden M. Morris

**Affiliations:** 1 Stanford-Surgery Policy Improvement Research and Education Center (S-SPIRE Center), Department of Surgery, Stanford School of Medicine, Palo Alto, CA, United States; 2 Department of Radiology, Stanford School of Medicine, Stanford, CA, United States; 3 Veterans Affairs Medical Center, Palo Alto, CA, United States; 4 Department of Radiation Oncology, Stanford University School of Medicine, Stanford, CA, United States

**Keywords:** rectal cancer, MRI, clinical complete response, deep learning, segmentation, nnU-Net

## Abstract

**Introduction:**

Traditional neoadjuvant therapy for locally advanced rectal cancer (LARC) results in pathologic complete response (pCR) in approximately 15% of patients, supporting non-operative strategies for those with clinical complete response (cCR). The subjectivity and variability in MRI-based cCR assessments highlight the need for objective, quantitative tools.

**Objective:**

To develop deep learning models for automated rectal tumor segmentation on pre- and post-treatment MRIs, and to identify radiomic features differentiating cCR from non-cCR patients.

**Materials and methods:**

We retrospectively analyzed pre- and post-treatment MRIs from 37 LARC patients enrolled in a Phase 2 TNT trial (NCT04380337). Rectal tumors were segmented on T2-weighted images by two data scientists, refined by a radiologist (reference standard), and independently segmented by a fellow. For pre-treatment segmentation, Model 1 (baseline; n=37) was trained on reference cases, then used to generate pseudo-labels for 81 additional cases. Model 2 (semi-supervised; n=118) was trained on the combined dataset. Model 3 (baseline; n=37) was trained on post-treatment cases. Radiomic features were extracted from post-treatment ADC maps, filtered by reproducibility (ICC ≥0.8) and redundancy (Spearman ρ≤0.95), then analyzed using unsupervised hierarchical clustering.

**Results:**

For pre-treatment segmentation, radiologist-fellow inter-rater agreement was DSC =0.748±0.092. Model 1 achieved mean DSC =0.682±0.254 versus the radiologist, significantly lower than inter-rater agreement. Model 2 improved performance to mean DSC =0.769±0.214 (mean gain =0.087; 12.8% relative improvement; p<0.001), slightly outperforming inter-rater agreement. For post-treatment segmentation, inter-rater agreement declined to mean DSC =0.362±0.256, while Model 3 achieved mean DSC =0.175±0.231 versus the radiologist, reflecting challenges from treatment-induced tissue changes affecting both automated models and human raters. Radiomic clustering revealed two distinct patient groups aligned with cCR and non-cCR status.

**Conclusion:**

This study demonstrates the feasibility of deep learning-based automated segmentation and radiomic profiling for differentiating treatment response in rectal cancer. Semi-supervised learning with pseudo-labeled data significantly improved segmentation performance, offering a practical approach to overcome limited annotations. Radiomic features warrant validation in larger multi-center studies for clinical translation.

## 1 Introduction

Traditional treatment for locally advanced rectal cancer (LARC) – neoadjuvant chemoradiation, radical surgery, and systemic chemotherapy – is effective but often debilitating. Surgical removal of the rectum incurs complication rates as high as 46% (1). Even without complications, approximately 25% of patients who undergo surgery require a colostomy, while the remaining 75% often struggle with bowel dysfunction, bladder control, and sexual health problems (2–4). In recent years, total neoadjuvant therapy (TNT) has emerged as a promising alternative, potentially enabling patients to avoid surgery altogether. After initial staging by pre-treatment magnetic resonance imaging (MRI), TNT consists of preoperative administration of radiation and full-dose systemic chemotherapy. Another MRI is then used to evaluate the clinical response to TNT. If the patient achieves a clinical complete response (cCR) – no evident tumor on clinical testing – they may be eligible for a “watch and wait” approach with close surveillance rather than surgery.

Due to its critical anatomical detail, MRI has become essential in the new paradigm of rectal cancer treatment and surveillance. In recent studies, up to 30–50% of patients who undergo TNT achieve a cCR and can safely avoid surgery (5–8). Accurate cCR assessment depends on the combined interpretation of MRI, endoscopy, and digital rectal examination (DRE), with MRI evaluation by the radiologist serving as a key determinant. The first step in interpretation involves manual segmentation of the rectal tumor by delineating the MRI of the tumor’s 3D volume of interest. Even among expert radiologists, however, manual segmentation can be inaccurate among up to 40% of cases (9) which can impact patient outcomes. Inter- and intra-rater variability, along with the challenges posed by indistinct tumor boundaries and the complexity of tumor morphology, underscores the need for standardized MRI protocols (10). An automated segmentation model tailored to rectal tumors on pre- and post-treatment MRI could enhance clinical decision-making, improve efficiency, and support radiologists’ interpretation, ultimately leading to better patient care in rectal cancer management.

Deep learning methods can automatically learn relevant features from medical images without manual feature engineering. We employed “no new U-Net” (nnU-Net) (11), a self-configuring deep learning framework that automatically adapts its architecture, pre-processing, and training procedures to dataset characteristics, eliminating manual hyperparameter tuning while achieving state-of-the-art segmentation performance. These automated segmentations then enable radiomic analysis, which extracts quantitative features capturing tumor intensity, texture, shape, and heterogeneity. The radiomic workflow includes feature extraction (first-order statistics, shape descriptors, and texture features) followed by statistical analysis to identify features associated with clinical outcomes. This approach provides objective, reproducible imaging biomarkers that can predict treatment response and support clinical decision-making.

Our long-term objective is to develop and validate an AI-driven model capable of reliably predicting cCR, thereby accurately identifying rectal cancer patients who can safely forgo surgery. In the current study, we aimed to take a critical step toward this objective by developing automated deep learning models, based on the nnU-Net framework (11), to segment rectal tumors on pre- and post-treatment MRIs.

Although automated segmentation already has demonstrated effectiveness in cancers such as lung, breast, and prostate, its application to rectal cancer has been limited by the scarcity of large-scale, annotated MRI datasets.

## 2 Materials and methods

### 2.1 Study cohort

**Figure 1:**
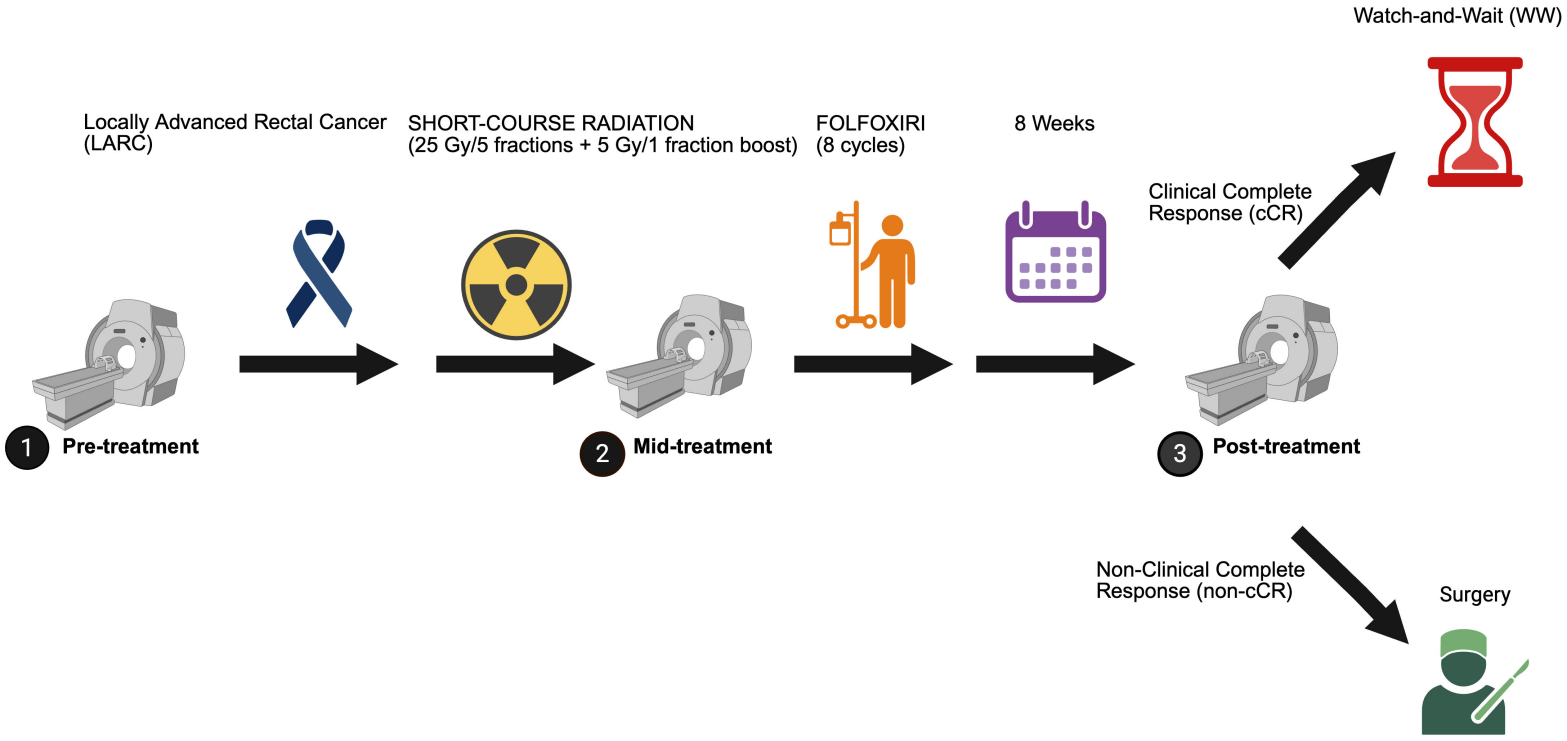
MRI timing during the treatment schema for the Phase 2 clinical trial of TNT in patients with LARC (NCT04380337). Created in BioRender. Selby, H. (2025) https://BioRender.com/frgsmrc.

We conducted a secondary analysis of MRI data from patients enrolled in a Phase 2 clinical trial of TNT for rectal cancer (**Figure 1**; NCT04380337), conducted between May 2020 and April 2023 (12). The study was approved by Stanford’s Institutional Review Board, (Protocol #: IRB-62555). This trial assessed the efficacy of combining short-course radiotherapy with chemotherapy (FOLFOXIRI) to enhance cCR rates and facilitate organ preservation.

### 2.2 Treatment protocol and clinical response

As previously reported (12), patients received short-course radiation therapy consisting of 25*Gy* in 5 daily fractions with a sequential 5*Gy* boost to gross disease (total 30*Gy* in 6 fractions), delivered via intensity-modulated radiation therapy (IMRT). Following a 2−4 week interval, patients received up to 8 cycles of FOLFOXIRI chemotherapy: oxaliplatin 85*mg/m*^2^, irinotecan 165*mg/m*^2^, leucovorin 200*mg/m*^2^, and 5-fluorouracil 3200*mg/m*^2^ continuous infusion over 48 hours, repeated every 14 days. Clinical response was evaluated 8 ± 4 weeks after chemotherapy completion using pelvic MRI with tumor regression grading (mrTRG), flexible sigmoidoscopy, and DRE. Clinical complete response (cCR) was defined as absence of residual tumor on endoscopy/DRE and mrTRG 1−2 on MRI. Patients achieving cCR were offered organ preservation with intensive surveillance including quarterly endoscopy, semi-annual MRI, and biannual CT imaging.

Among the 37 trial patients, 9 achieved cCR and 28 did not (**Supplementary Table S1**). The median age of the study cohort was 52 years (IQR: 45−61). Patients with cCR were significantly older (median 57 years, IQR: 54−65) than those without cCR (median 50 years, IQR: 41−58). The cCR group also contained more men (78%) than the non-cCR group (61%). Before treatment, 70% of all patients had tumors with poor prognostic features, including 70.3% T3 stage, 22% T4 stage, and only 8% T2 stage. Nodal staging was evenly distributed among patients who did and did not have cCR.

### 2.3 MRI protocol

MRIs were acquired at three time points: pre-treatment, mid-treatment, and post-treatment. Imaging was performed using a 3T MRI scanner (GE or Siemens) with the following sequences and parameters: 2D Fast Spin Echo T2WI: TR 4000*ms*, TE 100*ms*, 288×288 matrix, 3*mm* slice thickness, no inter-slice gap; and DWI: reduced field-of-view, 112×64 matrix, 24*cm* field-of-view, 6*mm* slice thickness, acquired at *b*-values of 50 and 800*s/mm*^2^. Consistent with best practice, patient preparation included administration of a micro-enema, application of 50−150*mL* rectal gel based on tumor location, and intravenous injection of 1*mg* IV glucagon prior to axial T2WI to reduce peristalsis.

### 2.4 Automated tumor segmentation using deep learning

**Figure 2:**
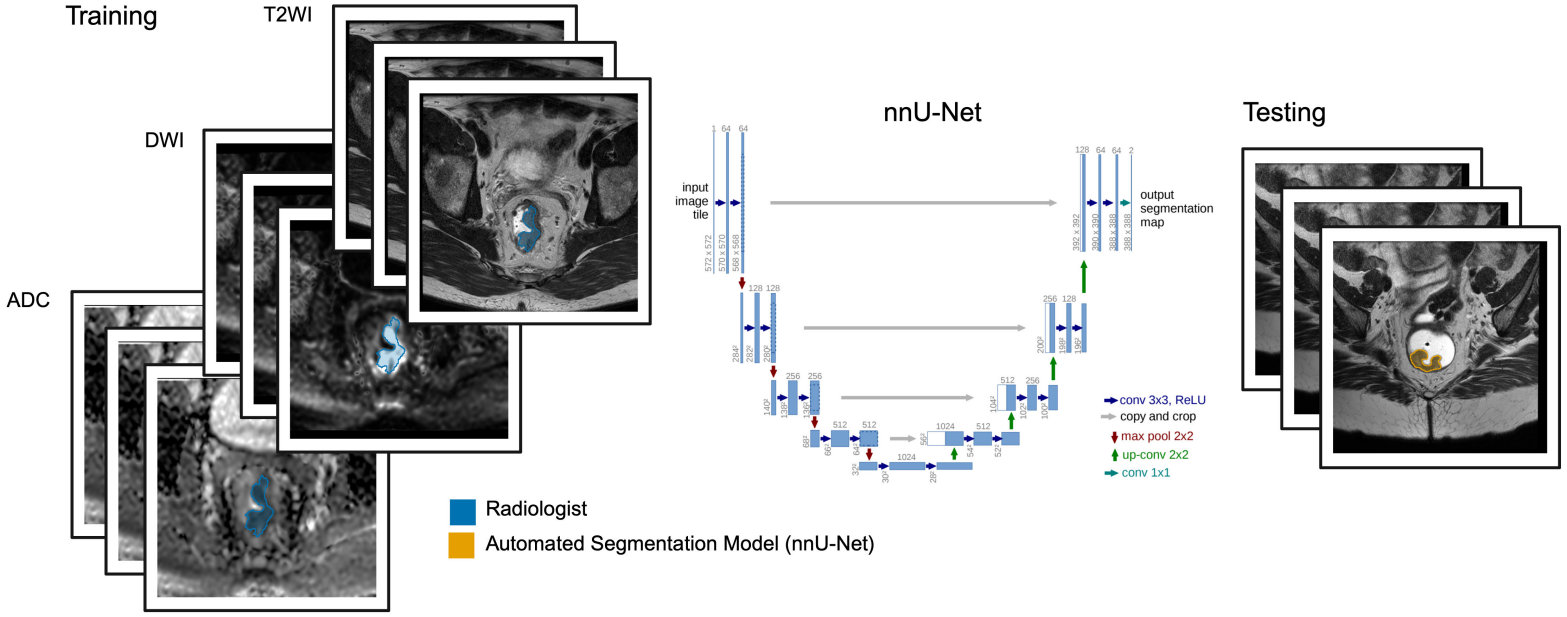
T2WIs, DWIs, and ADC maps from a Phase 2 clinical trial (n=37), along with rectal tumor segmentations delineated by a radiologist (blue) were used to train automated segmentation models (nnUNet) using 5-fold cross-validation. Three models were developed: Model 1 (baseline; trained on n=37 pre-treatment images), Model 2 (semi-supervised; trained on n=118 pre-treatment images including 81 pseudo-labeled cases generated by Model 1), and Model 3 (baseline; trained on n=37 post-treatment images). Example segmentation output from Model 1 is shown in orange. Created in BioRender. Selby, H. (2025) https://BioRender.com/frgsmrc.

We used the deep learning-based segmentation framework nnU-Net (11) (**Figure 2**). Three models were trained and validated using 5-fold cross-validation: Model 1 (baseline model trained on *n* = 37 pre-treatment MRIs), Model 2 (semi-supervised model trained on *n* = 118 pre-treatment images), and Model 3 (baseline model trained on *n* = 37 post-treatment images). All models used magnetic resonance imaging (MRI) sequences including T2-weighted images (T2WIs), synthetic diffusion-weighted images (sDWIs; *b*-value= 1500*s/mm*^2^), and apparent diffusion coefficient (ADC) maps, with corresponding manual tumor segmentations delineated by a radiologist and fellow on T2WIs. For pre-treatment MRI segmentation, we trained Model 1 on 37 reference standard cases, then used it to generate pseudo-labels for 81 additional cases. Model 2 was trained on the combined 118 cases (37 reference standard + 81 pseudo-labeled cases). For post-treatment MRI segmentation, we trained Model 3 on 37 reference standard cases.

### 2.5 Manual segmentation

Rectal tumors were manually segmented in 3D on pre- and post-treatment axial T2WIs using Slicer 5.8.0.13 (13). Initial contours were delineated slice-by-slice by two data scientists, which were then reviewed and refined by a U.S. board-certified radiologist to establish the reference standard (14). Additionally, a radiology fellow independently segmented all tumors to enable inter-rater variability assessment.

### 2.6 MRI and segmentation pre-processing

Each DWI was resampled to match the reference T2WI space using B-spline interpolation. This resampling ensured accurate spatial alignment, preserving anatomical details and tumor boundary fidelity, facilitating effective integration of multi-modal imaging data. Segmentation pre-processing included maximum connected volume selection, retaining only the largest contiguous voxel group, thus eliminating smaller, disconnected segments caused by artifacts or stray pixels. Additionally, hole filling was performed to include unsegmented regions completely enclosed within segmented areas, ensuring comprehensive and accurate tumor delineation. Imaging and segmentation pre-processing was done in Python v3.12.1 using SimpleITK v2.4.0.

### 2.7 Calculated ADC and sDWIs

ADC maps quantify water diffusion within tissues, derived from signal decay observed in DWIs, and are essential for tumor characterization. ADC maps were calculated using DWIs acquired at *b*-values of 0, 50, and 800*s/mm*^2^ based on the mono-exponential decay model (General model in **Equation 1**; our parameters applied to the model in **Equation 2**):

(1) $$S(b)=S_{0}.e^{\left(-bADC\right)}$$

where *S*(*b*) is signal intensity at a given *b*-value, *b* is diffusion weighting (*s/mm*^2^), and ADC is in *mm^/^s*. sDWIs at high *b*-value (*b* = 1500*s/mm*^2^) were generated from the calculated ADC maps to simulate diffusion contrast without direct acquisition:

(2) $$sDWI_{1500}=S_{0}.e^{\left(-1500.ADC\right)}$$

This approach enhances image quality and tumor conspicuity while reducing scan time and patient discomfort. This step was performed in Python v3.12.1 using SimpleITK v2.4.0.

### 2.8 Radiomic analysis

**Figure 3:**
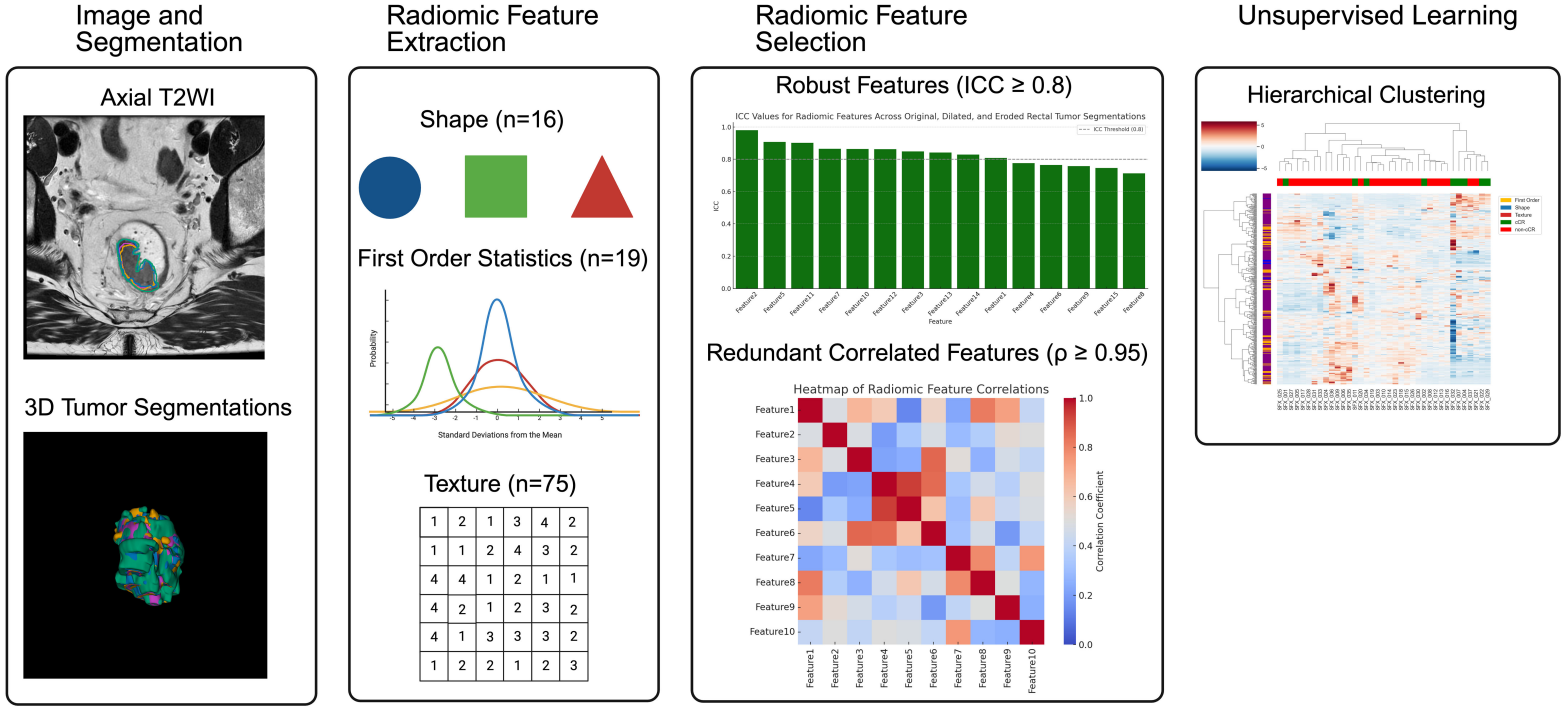
Radiomics workflow: 1) Image and Segmentation: Rectal tumors were manually segmented on axial T2WIs by a radiologist. 2) Radiomic Feature Extraction: A total of 1317 radiomic features were extracted from the corresponding quantitative ADC maps using the segmented rectal tumors. These included shape (n=16), first-order statistics (n=19), and texture features (n=75), derived from the original images, as well as from images processed with Laplacian of Gaussian (LoG, *σ* = 1–5) and wavelet filters. 3) Radiomic Feature Selection: Robust and reproducible features were retained based on intraclass correlation coefficient (ICC ≥ 0.8) and low inter-feature correlation (*ρ* ≤ 0.95). 4) Unsupervised Learning: Hierarchical clustering was performed to group patients based on radiomic feature similarity, independent of clinical labels. Created in BioRender. Selby, H. (2025) https://BioRender.com/ofq574j.

The radiomic feature extraction and analysis workflow is summarized in **Figure 3**. Features were extracted from post-treatment ADC maps using rectal tumor segmentations delineated by a radiologist on T2WIs. ADC was chosen for its quantitative nature compared to conventional T2WIs and DWIs. Features included shape (*n* = 16), first-order statistics (*n* = 19), and texture features (*n* = 75), derived from the original images as well as from filtered images using Laplacian of Gaussian (LoG, *σ* = 1.0−5.0) and wavelet decompositions. To emulate inter-rater variability and assess feature robustness, automated tumor segmentations were dilated and eroded. Robust features were retained based on high intra-class correlations (ICC ≥ 0.8), while redundant features showing high Spearman correlation (Spearman *ρ* ≥ 0.95) were discarded. Clinical and imaging features were clustered hierarchically and assessed for correlation with treatment outcomes (cCR versus non-cCR), based on chart review.

### 2.9 Statistical analysis

Sample size n=37 was calculated for the primary clinical endpoint (cCR rate) (12). For the current secondary automated segmentation, we enriched the training data to *n* = 118 through semi-supervised learning with pseudo-labeled data for an additional *n* = 81 cases, while maintaining the same *n* = 37 test cases for evaluation. We compared cCR and non-cCR groups using Wilcoxon rank-sum test for continuous variables (age) and chi-square or Fisher’s exact test for categorical variables (sex, tumor stage, nodal stage, tumor location, MRI manufacturer, and slice thickness). Dice Similarity Coefficients (DSCs) were calculated to assess pairwise agreement between segmentations from the radiologist, fellow, and three deep learning models: Model 1 (baseline model trained on *n* = 37 pre-treatment images), Model 2 (semi-supervised model trained on *n* = 118 pre-treatment images), and Model 3 (baseline model trained on *n* = 37 post-treatment images). For each comparison, we computed mean DSC ± standard deviation (SD) along with mean differences and bias-corrected and accelerated (BCa) bootstrapped 95% confidence intervals (CI) using 10,000 resamples. Statistical significance was assessed using both paired *t*-tests (assuming normally distributed differences) and Wilcoxon signed-rank tests (non-parametric alternative). Radiologist-fellow inter-rater agreement served as the reference standard for model performance evaluation. To evaluate the semi-supervised learning approach, we directly compared Model 2 versus Model 1 performance using paired statistical tests on the same *n* = 37 test cases. All statistical tests were 2-sided; *p <* 0.05 was considered statistically significant. Pre-processing, post-processing, calculations and data analysis were performed with Python v3.12.1.

### 2.10 Code availability

Code for training and inference, along with model hyper-parameters and weights, was developed using Python v3.12.1 and PyTorch v2.6. These resources are publicly available on the GitHub repository https://github.com/s-spire-research/Rectal-Tumor-MRI-SEG.

## 3 Results

### 3.1 MRI characteristics

Pre-treatment MRIs were acquired using scanners from GE Medical Systems (65%), Siemens (22%), and Philips (14%) (**Table 1**). Slice thickness was predominantly standardized at 3.0*mm* (92%), with a small number acquired at 2.5*mm* (5%) or 3.5*mm* (3%). Post-treatment MRIs were almost exclusively obtained using GE Medical Systems scanners, with a single scan performed on a Siemens system. All post-treatment MRIs were acquired with a slice thickness of 3.0*mm*.

**Table 1:** MRI acquisition parameters by clinical response group.

Timepoint	MRI characteristic	non-cCR (*n* = 28)	cCR (*n* = 9)	Total (*n* = 37)	*P*-value
Pre-Tx	MRI Manufacturer, n (%)				0.62
	GE Medical Systems	19 (68)	5 (56)	24 (65)	
	Philips	4 (14)	1 (11)	5 (14)	
	Siemens	5 (18)	3 (33)	8 (22)	
	Slice Thickness (mm), n (%)				0.59
	2.5	2 (7)	0	2 (5)	
	3.0	25 (89)	9 (100)	34 (92)	
	3.5	1 (4)	0	1 (3)	
Pre-Tx	MRI Manufacturer, n (%)				1.00
	GE Medical Systems	27 (96)	9 (100)	36 (97)	
	Siemens	1 (4)	0	1 (3)	
	Slice Thickness (mm), n (%)				1.00
	3.0	28 (100)	9 (100)	37 (100)	

Pre-Tx, pre-treatment; Post-Tx, post-treatment; cCR, clinical complete response; non-cCR, non-clinical complete response.

### 3.2 Automated segmentation (nnU-Net) performance

**Figure 4:**
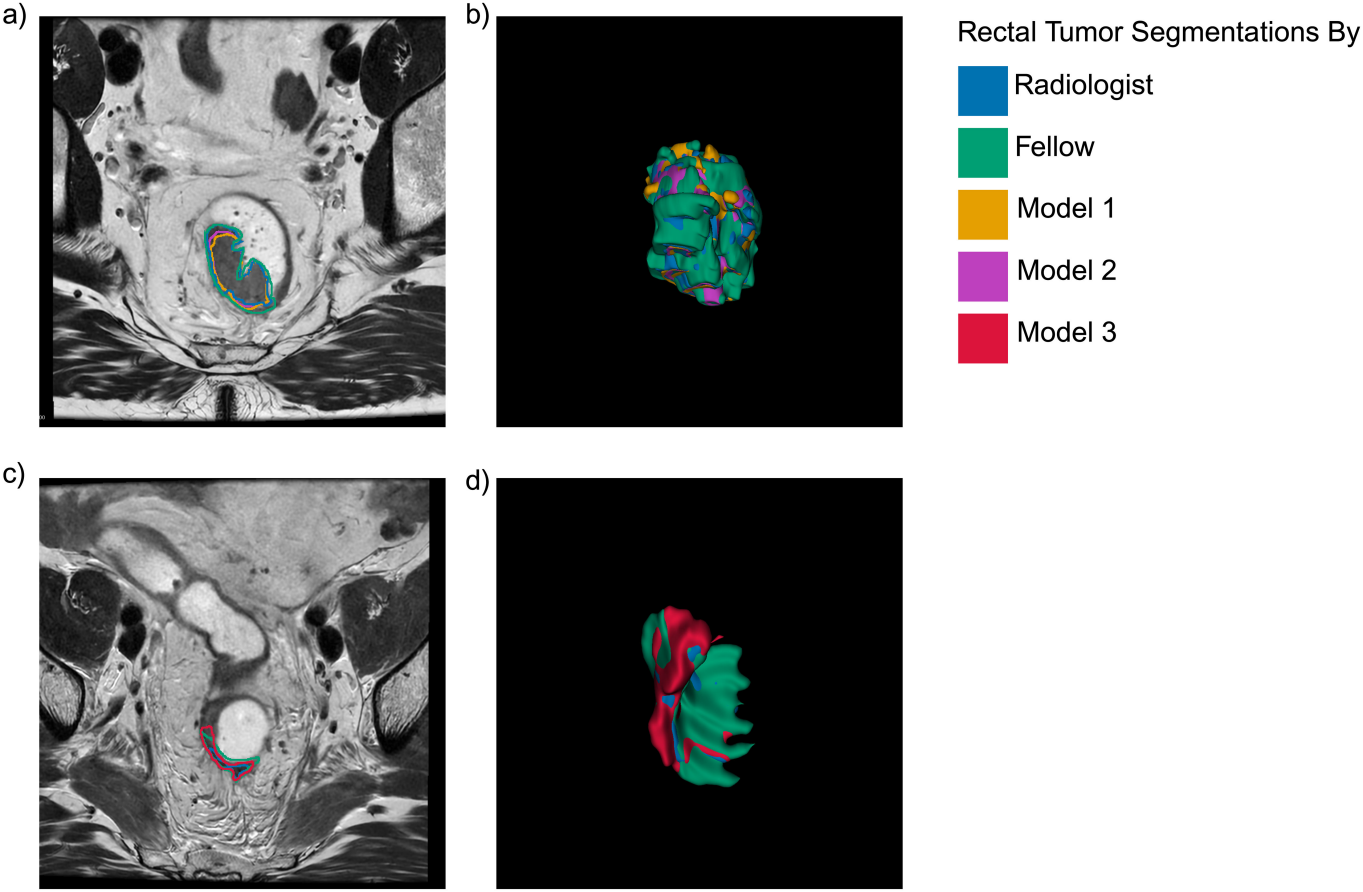
Pre-treatment **(a, b)** and post-treatment **(c, d)** rectal tumor segmentations for a representative patient who achieved complete clinical response. T2WIs **(a, c)** and corresponding 3D renderings **(b, d)** show manual segmentations by the radiologist (blue) and fellow (green), alongside automated nnU-Net predictions: Model 1 (orange), Model 2 (purple), and Model 3 (red). For this case, pre-treatment DSCs were: Model 1 vs. radiologist = 0.892, vs. fellow = 0.854; Model 2 vs. radiologist = 0.911, vs. fellow = 0.857. Post-treatment DSCs were: Model 3 vs. radiologist = 0.452, vs. fellow = 0.339. Inter-rater agreement was DSC = 0.858 pre-treatment and 0.652 post-treatment. Model 2 demonstrated superior pre-treatment overlap, while post-treatment segmentation (Model 3) remained challenging due to treatment-induced tissue changes. Created in BioRender. Selby, H. (2025) https://BioRender.com/ga308oy.

**Figure 4** illustrates representative rectal tumor segmentations for a patient who achieved cCR. In panels a and b (pre-treatment), both automated models showed strong agreement with manual segmentations: Model 1 (orange) achieved DSCs of 0.892 and 0.854 compared to the radiologist (blue) and fellow (green), respectively, while Model 2 (purple) achieved DSCs of 0.911 and 0.857. Post-treatment (panels c and d), Model 3 (red) achieved DSCs of 0.452 and 0.339 compared to the radiologist and fellow, respectively. Inter-rater agreement between the radiologist and fellow was DSC = 0.858 pre-treatment and declined to DSC = 0.652 post-treatment. This exemplar case illustrates the inherent difficulty of post-treatment tumor delineation, where residual fibrosis and treatment effects obscure tumor boundaries for human raters.

**Table 2** presents quantitative segmentation performance across all 37 patients. For pre-treatment segmentation, radiologist-fellow inter-rater agreement served as the reference standard with a mean DSC of 0.748 ± 0.092. Model 1 (baseline; *n* = 37) achieved mean DSCs of 0.682 ± 0.254 when compared to the radiologist and 0.639 ± 0.249 when compared to the fellow, both significantly lower than the inter-rater benchmark. Model 1 underperformed both raters, with mean differences of −0.067 vs. the radiologist (95% BCa CI: −0.16 to 0.001; Wilcoxon *p* = 0.79) and −0.11 vs. the fellow (95% BCa CI: −0.20 to −0.047; paired *t*-test *p* = 0.007, Wilcoxon *p* = 0.042). Model 2 (semi-supervised; *n* = 118) demonstrated improved performance with mean DSCs of 0.769 ± 0.214 compared to the radiologist and 0.687 ± 0.201 compared to the fellow. Notably, Model 2 slightly outperformed the fellow (mean difference: +0.021, 95% BCa CI: −0.073 to 0.076), though statistical significance varied by test (paired *t*-test *p* = 0.58, Wilcoxon *p* = 0.008). These discordant results between paired t-test (*p* = 0.58) and Wilcoxon test (*p* = 0.008) suggest non-normal distribution of differences, with the non-parametric Wilcoxon test considered more reliable. Model 2 showed a mean difference of −0.061 (95% BCa CI: −0.14 to −0.015; paired *t*-test *p* = 0.060, Wilcoxon *p* = 0.29) compared to the fellow.

**Table 2:** Dice similarity coefficients comparing radiologist, fellow, and automated models (*n* = 37 test cases).

Timepoint	Comparison	Mean DSC	Difference	*t*-test	Wilcoxon
		(SD)	[95% BCa CI]	*p*-value*^a^*	*p*-value*^b^*
Pre-Tx	Radiologist vs Fellow*^c^*	0.748 (0.092)	–	–	–
	Model 1 vs Radiologist	0.682 (0.254)	−0.067 [−0.16 to 0.001]	0.11	0.79
	Model 1 vs Fellow	0.639 (0.249)	−0.11 [−0.20 to −0.047]	0.007	0.042
	Model 2 vs Radiologist	0.769 (0.214)	+0.021 [−0.073 to 0.076]	0.58	**0**.**008**
	Model 2 vs Fellow	0.687 (0.201)	−0.061 [−0.14 to −0.015]	0.060	0.29
Post-Tx	Radiologist vs Fellow*^d^*	0.362 (0.256)	–	–	–
	Model 3 vs Radiologist	0.175 (0.231)	−0.187 [−0.283 to −0.079]	*<* 0.001	*<* 0.001
	Model 3 vs Fellow	0.125 (0.199)	−0.237 [−0.322 to −0.159]	*<* 0.001	*<* 0.001

Pre-Tx, pre-treatment; Post-Tx, post-treatment; DSC, Dice similarity coefficient; SD, standard deviation; CI, confidence interval; BCa, bias-corrected and accelerated bootstrap with 10,000 resamples. Model 1 = baseline model trained on pre-treatment images (*n* = 37); Model 2 = semi-supervised model trained on pre-treatment images (*n* = 118); Model 3 = baseline model trained on post-treatment images (*n* = 37). For model comparisons, mean difference calculated as Model DSC − Radiologist/Fellow DSC; positive values indicate model outperformed the reference rater, negative values indicate model underperformed.

Significant values indicating improved model performance (*p <* 0.05) are bolded.

a Paired *t*-test assumes normally distributed differences.

b Wilcoxon signed-rank test is non-parametric (no normality assumption).

c Inter-rater agreement (reference standard) pre-treatment; DSC reported without statistical comparison.

d Inter-rater agreement (reference standard) post-treatment; DSC reported without statistical comparison.

Post-treatment segmentation proved more challenging for both Model 3 and expert raters. Model 3 (baseline; *n* = 37) achieved mean DSCs of 0.175 ± 0.231 versus the radiologist and 0.125 ± 0.199 versus the fellow, both significantly lower than post-treatment inter-rater agreement (mean differences: −0.187, 95% BCa CI: −0.283 to −0.079; and −0.237, 95% BCa CI: −0.322 to −0.159; both *p <* 0.001 for paired *t*-test and Wilcoxon test). Radiologist-fellow inter-rater agreement also declined from 0.748 ± 0.092 pre-treatment to 0.362 ± 0.256 post-treatment, underscoring again the inherent difficulty of post-treatment tumor delineation due to treatment-induced changes that challenged both human raters and automated models.

**Table 3** shows the direct comparison between Model 1 (baseline; *n* = 37) and Model 2 (semi-supervised; *n* = 118). Model 2 demonstrated an absolute mean gain of 0.087 (95% BCa CI: 0.052 to 0.132; 12.8% relative improvement; paired *t*-test *p <* 0.001, Wilcoxon *p <* 0.001) in agreement with the radiologist, improving in 36 of 37 cases (97.3%). The improvement in agreement with the fellow was more modest (mean gain: 0.049, 95% BCa CI: 0.019 to 0.085; 7.6% relative improvement; paired *t*-test *p <* 0.01, Wilcoxon *p <* 0.05), with 22 of 37 cases (59.5%) showing improvement and 15 (40.5%) showing decreased agreement, demonstrating that Model 2 primarily enhanced consistency with the radiologist’s segmentation approach.

**Table 3:** Direct comparison of model 2 versus model 1 performance (*n* = 37 cases).

Reference	Mean gain	95% BCa CI	Paired *t*-test	Wilcoxon	Improved
rater	(Relative)	[Difference]	*p*-value	*p*-value	cases
Radiologist	0.087 (12.8%)	[0.052 to 0.132]	*<***0**.**001**	*<***0**.**001**	36*/*37 (97.3%)
Fellow	0.049 (7.6%)	[0.019 to 0.085]	*<***0**.**01**	*<***0**.**05**	22*/*37 (59.5%)

BCa. bias-corrected and accelerated bootstrap with 10,000 resamples. Mean gain calculated as Model 2 DSC − Model 1 DSC. Model 1 = baseline model trained on *n* = 37 images; Model 2 = semi-supervised model trained on *n* = 118 images. Both models evaluated on the same 37 cases. Significant values (*p <* 0.05) are bolded.

### 3.3 Radiomics analysis

A total of 1317 radiomic features were extracted from ADC maps and the rectal tumor segmentations delineated by the radiologist on T2WIs. We identified 416 highly reliable radiomic features (ICC ≥ 0.8) with minimal redundancy (Spearman *ρ* ≤ 0.95), indicating robust reproducibility and relevance for clinical interpretations. We performed hierarchical clustering using these 416 robust and reproducible radiomic features, after applying z-score standardization to normalize feature scales across patients.

**Figure 5:**
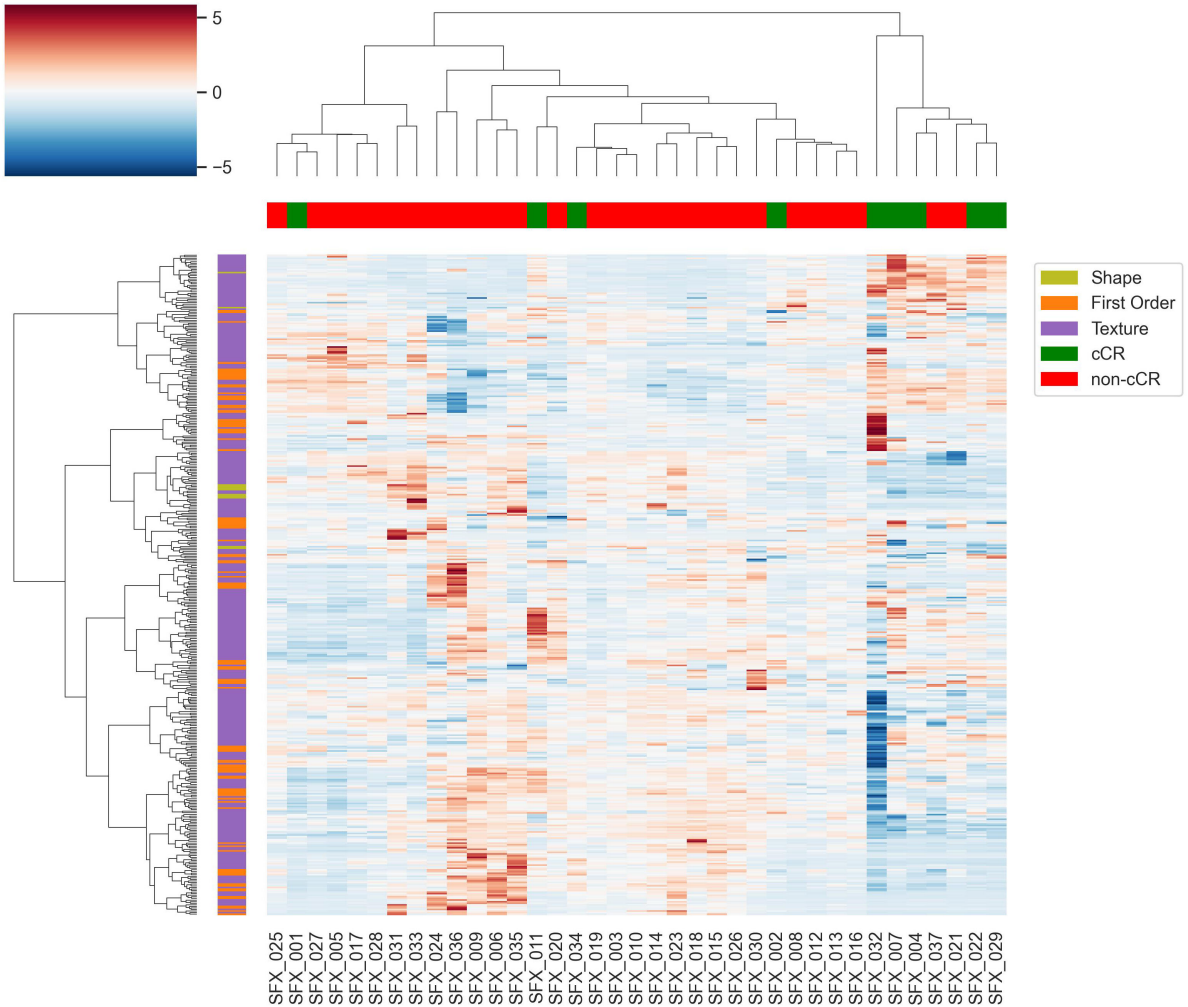
Hierarchical clustering heatmap of radiomic features extracted from rectal tumor segmentations on ADC images. Rows represent radiomic features—shape, first-order, and texture—color-coded at left (blue: shape; orange: first-order; red: texture). Columns represent individual patients, with cCR status indicated (green: cCR; red: non-cCR). Clustering was performed after z-score standardization and selection of 416 highly reliable radiomic features (ICC ≥ 0.8) with minimal redundancy (pairwise correlation ≤ 0.9), indicating robust reproducibility and clinical relevance. Heatmap colors reflect z-scored feature values (red = high, blue = low). Notable cases: SFX 002 had tumor regrowth, and SFX 011 and SFX 029 had a pathologic complete response following abdominoperineal resection (APR).

**Figure 5** presents a hierarchical clustering heatmap of radiomic features extracted from quantitative post-treatment ADC maps using radiologist-delineated rectal tumor segmentations. Each row represents a radiomic feature, color-coded by category (blue: shape, orange: first-order, red: texture), and each column corresponds to an individual patient. Clustering was performed based solely on radiomic feature similarity, without knowledge of clinical outcomes. Despite this, the resulting clusters show clear alignment with clinical response: patients who achieved a cCR are highlighted in green, while those without cCR are shown in red. Two distinct patient clusters emerged, suggesting strong intrinsic differences in imaging phenotypes between response groups.

We also noted the following 3 outliers based on chart review after post-treatment MRI: SFX 002, who experienced tumor regrowth; SFX 011 and SFX 029, who had a pathologic complete response following abdominoperineal resection (APR). These examples further support the concordance between radiomic clustering and meaningful clinical outcomes.

## 4 Discussion

In this secondary analysis of data from a Phase 2 clinical trial of TNT for rectal cancer, we developed three nnU-Net models to automate rectal tumor segmentation on pre- and post-treatment MRIs. Model 1 (baseline; *n* = 37) achieved lower agreement with both expert raters than the raters achieved with each other, demonstrating the challenge of training deep learning models with limited annotated data. Model 2 (semi-supervised; *n* = 118) substantially improved performance, achieving agreement levels that matched or slightly exceeded radiologist-fellow inter-rater agreement. The improvement was greater in agreement with the radiologist (97.3% of cases) than the fellow (59.5%), as Model 1, trained on the radiologist’s segmentations, generated the pseudo-labels for the additional 81 cases used in Model 2. This reinforced the radiologist’s segmentation approach, demonstrating that pseudo-labeled data effectively captures and propagates the expert segmentation patterns from which they originate, offering a practical solution to limited annotations in medical imaging.

Post-treatment segmentation (Model 3) proved more challenging due to treatment-induced changes such as fibrosis, edema, and scar tissue. Model 3 achieved lower agreement with each rater than the raters achieved with each other, though radiologist-fellow inter-rater agreement itself declined from pre-treatment levels, with notably increased variability. This parallel decline in both human and automated performance highlights that this is not solely a model limitation but a fundamental imaging challenge affecting both automated algorithms and human raters. The decline underscores the biological complexity of treatment response assessment, where tumor boundaries become indistinct due to treatment-induced tissue changes such as fibrosis, edema, and tissue remodeling. Despite these post-treatment segmentation challenges, radiomic features extracted from the radiologist’s segmentations still showed distinct imaging profiles associated with cCR versus non-cCR outcomes, demonstrating potential as predictive biomarkers for treatment response and suggesting that quantitative analysis may capture meaningful signal even when visual delineation is difficult.

Leveraging deep learning-based automated segmentation models further facilitates the extraction of robust radiomic features from MRI data, enhancing their applicability as predictive biomarkers. While the use of radiomics is well-established in breast, lung, and prostate cancers (15–19), MRI-based radiomics in rectal cancer remains relatively novel. Our study demonstrates the utility and feasibility of MRI-based radiomics in rectal cancer, underscoring its potential to standardize MRI interpretation, reduce subjectivity among radiologists, and refine criteria for assessing treatment response. In particular, radiomic features derived from post-treatment segmentations and ADC maps effectively differentiated between cCR and non-cCR patients, supporting clinical decision-making and personalized management strategies. Continued radiomic analyses, especially those incorporating pre- and post-treatment segmentation, are essential for refining predictive accuracy and optimizing patient selection for non-operative management strategies as TNT becomes more widely adopted in rectal cancer management.

In addition to its potential utility in clinical care, automated segmentation models could improve the efficiency of radiomic studies with further refinement, as these typically depend on manual segmentation by multiple radiologists. Leveraging deep learning-based approaches can reduce the manual annotation workload, enabling scalable analysis of larger datasets and multi-institutional studies. However, due to suboptimal performance in post-treatment rectal tumor segmentation (Model 3), continued radiologist oversight remains essential, particularly in challenging post-treatment scenarios. Future efforts should focus specifically on improving post-treatment segmentation through larger datasets, potentially employing similar semi-supervised learning strategies that proved successful for pre-treatment segmentation, and developing specialized architectures optimized for post-treatment anatomy.

This study has several limitations. First, the small sample size of reference standard annotations limits generalizability, though we mitigated this through semi-supervised learning with additional pseudo-labeled cases (Model 2) for pre-treatment segmentation. While our study cohort enabled initial model development and identified promising radiomic signatures, validation in larger, multi-institutional datasets is essential before clinical implementation. Second, post-treatment segmentation performance declined, reflecting the inherent complexity of post-treatment anatomy where tumors are obscured by radiation-induced fibrosis, edema, and scar tissue. Importantly, inter-rater agreement also declined post-treatment, highlighting this is not solely a model limitation but a fundamental imaging challenge. Future work should prioritize post-treatment segmentation accuracy through: (1) applying semi-supervised learning approaches similar to those successful for pre-treatment segmentation, (2) training on larger post-treatment datasets, (3) incorporating longitudinal imaging to track changes, and (4) developing specialized architectures for post-treatment anatomy. Until these improvements are achieved, radiologist oversight remains essential for post-treatment assessments.

Third, nnU-Net models were trained on segmentations delineated by a radiologist. While we used morphological operations (erosion/dilation) to simulate inter-rater variability for robustness testing, we acknowledge this may not capture the full spectrum of clinical variability. The pseudo-labels generated by Model 1 inherently contain errors from the baseline model, yet training on this noisy data (Model 2) still improved performance, suggesting semi-supervised learning is robust to label imperfections. Model 2 demonstrated greater improvement in agreement with the radiologist compared to the fellow, suggesting the model may have learned the radiologist’s specific segmentation approach rather than achieving universal improvement across all segmentation styles. This highlights that semi-supervised learning performance may be influenced by the characteristics of the initial training data and the source of pseudo-labels. Future studies should incorporate annotations from multiple radiologists to better represent clinical variability and establish more robust reference standard segmentations. Fourth, although rectal tumor segmentations were performed on T2WIs, nnU-Net training was conducted on sDWIs and ADC maps, while radiomic feature extraction was performed solely on ADC maps without prior image registration. This spatial mismatch between the segmentation masks and the underlying DWI and ADC map data may introduce inaccuracies and affect both model performance and feature reliability. We observed that conventional rigid registration was insufficient to align T2WIs, ADC maps, and sDWIs due to anatomical and acquisition differences, particularly in the pelvis.

Finally, although we incorporated T2WIs, ADC maps, and high *b*-value sDWIs into the nnU-Net framework as a three-channel input, the model treats all three modalities equally. This may not reflect the varying diagnostic value of each sequence, and future work could explore modality-specific weighting or attention mechanisms to optimize multi-modal integration. As a single-center secondary analysis, our findings require external validation. The specific patient population (primarily with high-risk features), treatment protocol (dose-escalated SCRT with FOLFOXIRI), and imaging protocols may not generalize to other centers or treatment approaches. Multi-institutional validation with diverse protocols and patient populations is essential before clinical translation. Despite these limitations, our study provides important proof-of-concept that automated segmentation with semi-supervised learning and radiomics can differentiate treatment response in rectal cancer, warranting larger validation studies.

## 5 Conclusion

We have demonstrated the feasibility and potential of integrating deep learning-based automated segmentation and ADC-based radiomics to enhance evaluation of rectal cancer treatment response. By employing a semi-supervised learning approach, we successfully addressed the challenge of limited training data – a pervasive barrier in medical imaging AI. Model 2 (semi-supervised) improved performance compared to Model 1 (baseline trained on limited data), achieving agreement levels that approached or slightly exceeded inter-rater variability. This finding offers a practical and scalable strategy for developing robust deep learning models in annotated data-scarce clinical scenarios. While challenges remain in post-treatment segmentation, where both automated models and human raters face difficulties due to treatment-induced tissue changes, our results establish a foundation for future improvements. Ongoing refinements through semi-supervised learning with larger datasets, multi-radiologist validation, specialized post-treatment architectures, and standardized imaging protocols hold promise for enhancing automated segmentation accuracy. Such advancements have the potential to improve personalized clinical decision-making, enhance predictive accuracy, and optimize patient management strategies, ultimately facilitating safe and effective non-operative approaches in rectal cancer management.

## Data Availability

The MRI data utilized in this study contains Protected Health Information (PHI) and is subject to HIPAA regulations, which prohibit public sharing to safeguard patient privacy. De-identified data may be made available upon reasonable request, subject to approval by the appropriate IRB or ethics committee, in accordance with HIPAA guidelines. Requests to access these datasets should be directed to selbyh@stanford.edu.

## Supplementary material

Supplementary Table 1 Patient characteristics from SFX trial (12). IQR, Interquartile Range; cCR, clinical complete response; non-cCR, non-clinical complete response; Pre-tx, pre-treatment; Post-tx, post-treatment. Significant values (*p <* 0.05) are bolded.

## References

[1] Gamboa AC Lee RM Turgeon MK Varlamos C Regenbogen SE Hrebinko KA . Impact of postoperative complications on oncologic outcomes after rectal cancer surgery: an analysis of the US rectal cancer consortium. Ann Surg Oncol. (2021) 28:1712–21. doi: 10.1245/s10434-020-08976-8, PMID: 32968958 PMC7955158

[2] Kim S Kim MH Oh JH Jeong S Park KJ Oh H . Predictors of permanent stoma creation in patients with mid or low rectal cancer: results of a multicentre cohort study with preoperative evaluation of anal function. Colorectal Dis. (2020) 22:399–407. doi: 10.1111/codi.14898, PMID: 31698537

[3] Robitaille S Maalouf MF Penta R Joshua TG Liberman AS Fiore JF . The impact of restorative proctectomy versus permanent colostomy on health-related quality of life after rectal cancer surgery using the patient-generated index. Surgery. (2023) 174:813–8. doi: 10.1016/j.surg.2023.06.033, PMID: 37495462

[4] Rivard SJ Vitous CA Bamdad MC Lussiez A Anderson MS Varlamos C . I Wish There had been Resources”: A Photo-Elicitation Study of Rectal Cancer Survivorship Care Needs. Ann Surg Oncol. (2023) 30:3530–7. doi: 10.1245/s10434-022-13042-6, PMID: 36847958 PMC10460498

[5] Langenfeld SJ Davis BR Vogel JD Davids JS Temple LKF Cologne KG . The American society of colon and rectal surgeons clinical practice guidelines for the management of rectal cancer 2023 supplement. Dis Colon Rectum. (2024) 67:18–31. doi: 10.1097/DCR.0000000000003057, PMID: 37647138

[6] Rettig RL Beard BW Ryoo JJ Kulkarni S Gulati M Tam M . Total neoadjuvant therapy significantly increases complete clinical response. Dis Colon Rectum. (2023) 66:374–82. doi: 10.1097/DCR.0000000000002290, PMID: 35239525

[7] Erozkan K Elamin D Tasci ME Liska D Valente MA Alipouriani A . Evaluating complete response rates and predictors in total neoadjuvant therapy for rectal cancer. J Gastrointest Surg: Off J Soc Surg Aliment Tract. (2024) 28:1605–12. doi: 10.1016/j.gassur.2024.07.015, PMID: 39067745

[8] Asare E Venner E Batchelor H Sanders J Kunk P Hedrick T . Outcomes associated with total neoadjuvant therapy with non-operative intent for rectal adenocarcinoma. Front Oncol. (2024) 14:1374360. doi: 10.3389/fonc.2024.1374360, PMID: 39156701 PMC11328831

[9] Siddiqui MRS Bhoday J Battersby NJ Chand M West NP Abulafi A-M . Defining response to radiotherapy in rectal cancer using magnetic resonance imaging and histopathological scales. World J Gastroenterol. (2016) 22:8414–34. doi: 10.3748/wjg.v22.i37.8414, PMID: 27729748 PMC5055872

[10] Delli Pizzi A Basilico R Cianci R Seccia B Timpani M Tavoletta A . Rectal cancer MRI: protocols, signs and future perspectives radiologists should consider in everyday clinical practice. Insights into Imaging. (2018) 9:405–12. doi: 10.1007/s13244-018-0606-5, PMID: 29675627 PMC6108973

[11] Isensee F Jaeger PF Kohl SAA Petersen J Maier-Hein KH . nnU-Net: a self-configuring method for deep learning-based biomedical image segmentation. Nat Methods. (2021) 18:203–11. doi: 10.1038/s41592-020-01008-z, PMID: 33288961

[12] Klebaner D Brown E Fisher GA Shelton A Johnson TP Shaheen S . Phase II trial of organ preservation program using short-course radiation and FOLFOXIRI for rectal cancer (SHORT-FOX): Two-Year primary outcome analysis. Radiother Oncol. (2025) 207:110884. doi: 10.1016/j.radonc.2025.110884, PMID: 40209856

[13] Fedorov A Beichel R Kalpathy-Cramer J Finet J Fillion-Robin J-C Pujol S . 3D Slicer as an image computing platform for the Quantitative Imaging Network. Magnetic Resonance Imaging. (2012) 30:1323–41. doi: 10.1016/j.mri.2012.05.001, PMID: 22770690 PMC3466397

[14] Selby HM Son YA Sheth VR Wagner TH Pollom EL Morris AM . AI-ready rectal cancer MR imaging: a workflow for tumor detection and segmentation. BMC Med Imaging. (2025) 25:88. doi: 10.1186/s12880-025-01614-3, PMID: 40087634 PMC11909848

[15] Wang S Wei Y Li Z Xu J Zhou Y . Development and validation of an MRI radiomics-based signature to predict histological grade in patients with invasive breast cancer. Breast Cancer (Dove Med Press). (2022) 14:335–42. doi: 10.2147/BCTT.S380651, PMID: 36262333 PMC9574565

[16] You C Su G-H Zhang X Xiao Y Zheng R-C Sun S-Y . Multicenter radio multiomic analysis for predicting breast cancer outcome and unravelling imaging-biological connection. NPJ Precis Oncol. (2024) 8:193. doi: 10.1038/s41698-024-00666-y, PMID: 39244594 PMC11380684

[17] Selby HM Mukherjee P Parham C Malik SB Gevaert O Napel S . Performance of alternative manual and automated deep learning segmentation techniques for the prediction of benign and Malignant lung nodules. J Med Imaging. (2023) 10:1–14. doi: 10.1117/1.JMI.10.4.044006, PMID: 37564098 PMC10411216

[18] Shah RP Selby HM Mukherjee P Verma S Xie P Xu Q . Machine learning radiomics model for early identification of small-cell lung cancer on computed tomography scans. JCO Clin Cancer Inf. (2021) 5:746–57. doi: 10.1200/CCI.21.00021, PMID: 34264747 PMC8812622

[19] Yang F Ford JC Dogan N Padgett KR Breto AL Abramowitz MC . Magnetic resonance imaging (MRI)-based radiomics for prostate cancer radiotherapy. Trans Androl Urol. (2018) 7:445–58. doi: 10.21037/tau.2018.06.05, PMID: 30050803 PMC6043736

