# Supplementary material for: Deep learning analysis of MRI to assess rectal cancer treatment
Source: Front Oncol. 2026 Feb 9;15:1643852. doi: 10.3389/fonc.2025.1643852 (PMC12927481; doi:10.3389/fonc.2025.1643852)
Supplement: Supplementary Table 1 — Patient characteristics from SFX trial (12). IQR, Interquartile Range; cCR, clinical complete response; non-cCR, non-clinical complete response; Pre-tx, pre-treatment; Post-tx, post-treatment. Significant values (p < 0.05) are bolded. [file DataSheet1.pdf]

Table S1. Patient Characteristics from SFX Trial

Characteristic	non-cCR (n=28)	cCR (n=9)	Total (n=37)	*p*-value
Age, median [IQR], years	50 [41−58]	57 [54−65]	52 [45−61]	0.06
Sex, n (%)				0.45
Male	17 (61)	7 (78)	24 (65)	
Female	11 (39)	2 (22)	13 (35)	
Pre-tx Clinical T Stage, n (%)				0.66
T2	2 (7)	1 (11)	3 (8)	
T3	19 (68)	7 (78)	26 (70)	
T4	7 (25)	1 (11)	8 (22)	
Pre-tx Clinical N Stage, n (%)				0.23
N0	7 (25)	0	7 (19)	
N1	11 (39)	4 (44)	15 (41)	
N2	10 (36)	5 (56)	15 (41)	
Post-tx Clinical T Stage, n (%)				**< 0.001**
T0	1 (4)	9 (100)	10 (27)	
T1	1 (4)	0	1 (3)	
T2	8 (29)	0	8 (22)	
T3	15 (54)	0	15 (41)	
T4	3 (11)	0	3 (8)	
Post-tx Clinical N Stage, n (%)				0.22
N0	18 (64)	9 (100)	27 (73)	
N1	7 (25)	0	7 (19)	
N2	1 (4)	0	1 (3)	
N+	2 (7)	0	2 (5)	
Tumor Location, n (%)				0.05
Low	9 (32)	7 (78)	16 (43)	
Mid	14 (50)	1 (11)	15 (41)	
High	5 (18)	1 (11)	6 (16)	

Note. IQR: Interquartile Range; cCR: clinical complete response; non-cCR: non-clinical complete response. Pre-tx: pre-treatment; Post-tx: post-treatment. Significant values ($\boldsymbol{p < 0.05}$) are bolded.
